# Catalysis by subsurface metal atoms in Al(001) through electron transfer

**DOI:** 10.1093/nsr/nwae077

**Published:** 2024-02-29

**Authors:** Bo Sun, Ding Ma

**Affiliations:** Beijing National Laboratory for Molecular Science, New Cornerstone Science Laboratory, College of Chemistry and Molecular Engineering, Peking University, China; Beijing National Laboratory for Molecular Science, New Cornerstone Science Laboratory, College of Chemistry and Molecular Engineering, Peking University, China

Heterogeneous catalysis plays a crucial role in the chemical industry, which has evolved from the investigation of chemisorption to an electronic structure [1]. Diverse strategies, such as alloying, doping, and confining, have been applied to enhance the catalytic properties of active sites through electronic modulation. The electron transfer between active sites and substrates profoundly influences catalytic activity [2]. However, the identification of electrons in catalytic processes is generally challenging due to the difficulties in tracing dynamic processes. Thus, verifying metallic catalysis transferred with electron or electron interaction as media holds great significance.

Recently, Professor Landong Li from Nankai University and colleagues reported unique metallic systems containing buried catalytically active transitional metals and exposed catalytically inert main group metals [3], which exhibited remarkable performance in a variety of reactions such as alkyne semi-hydrogenation, olefin hydroformylation and Suzuki-coupling reactions, revealing the effective conduction of catalytic properties through metallic bonding. Inspired by the disappearance of surface transitional metal species on aluminum substrate upon annealing, density functional theory (DFT) calculations were conducted by the team. It was observed that several *d*-block metals showed a tendency to self-disperse and sink, and could be well stabilized in the subsurface region of single crystal aluminum. Meanwhile, the electronic interaction between buried single transitional metal and adjacent aluminum atoms via metallic bonding was well demonstrated.

Based on the results of these calculations, they began to seek solid evidence on the structure of the unique metallic system (M/Al) and the electron transfer between transitional metals and aluminum substrate through scanning tunneling microscopy (STM). Typically, it was found that both palladium and rhodium exclusively located in the subsurface region of Al(001) and Al(111) single crystals as isolated atoms, with distinct electron transfer from palladium or rhodium to adjacent aluminum atoms.

**Figure 1.**
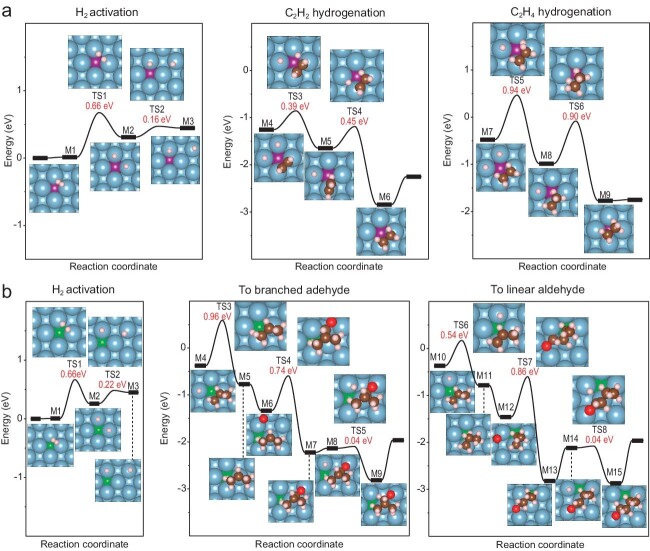
The semi-hydrogenation of acetylene and propylene hydroformylation over Pd/Al and Rh/Al in the conductive catalytic system, respectively, with Pd or Rh atoms completely buried in the subsurface of Al substrate [3].

The research team then performed DFT predictions on the catalytic properties of M/Al in several important reactions, including acetylene semi-hydrogenation and propylene hydroformylation (Fig. 1). The results revealed that the intrinsic catalytic properties of palladium and rhodium could be conducted to the outermost inert aluminum layer, yielding catalytically active M/Al systems, even though palladium and rhodium atoms were completely buried inside the aluminum single crystal and inaccessible to reaction substrates.

Guided by theoretical predictions and surface science observations, the team finally constructed real main group metal systems containing buried transitional metal centers, which showed excellent catalytic performance in reactions of alkyne semi-hydrogenation, olefin hydroformylation and Suzuki-coupling. These results further confirm that the catalytic properties of buried transitional metals can be transferred to exposed catalytically inert main group metals, thereby illustrating the conductive catalysis.

In contrast to traditional supported systems, this concept might offer an efficient shield for conventional active centers against poisoning or leaching by the conductive layer. More importantly, it is proposed that the catalytic properties of buried transitional metals can be precisely regulated or completely altered when passing through the conductive layer.
